# Motion perception with visual prostheses

**DOI:** 10.1088/2516-1091/ae46fd

**Published:** 2026-04-08

**Authors:** Kai T Renshaw, John S Pezaris

**Affiliations:** 1 Tufts University School of Medicine, Boston, MA, United States of America; 2 Department of Neurosurgery, Massachusetts General Hospital, Boston, MA, United States of America; 3 Department of Neurosurgery, Harvard Medical School, Boston, MA, United States of America

**Keywords:** artificial vision, phosphenes, frontoparallel motion, looming detection, gaze contingency

## Abstract

Visual prostheses represent a groundbreaking avenue for restoring vision in individuals with visual impairments. These devices utilize electrode arrays positioned in early visual processing areas, like the retina, thalamus, or primary visual cortex. Connected to a camera, they transform a stream of video to electrical stimulation to present the visual environment through patterned activation of phosphenes. Visual prostheses offer the potential to enhance visual function and thereby quality of life for users, however, understanding and replicating motion perception in a manner akin to natural vision remains a critical challenge for device designers. This review presents studies of motion perception in different visual prosthesis modalities and discusses their advantages and limitations. Retinal and cortical visual prostheses show significant potential in enhancing motion perception, but many implementations have shortcomings. Some challenges which remain for better motion perception in visual prosthesis are gaze contingency, the effective integration of machine vision, and understanding the involvement of higher-order visual areas. Despite these challenges, the current research should be viewed with substantial optimism for the future of restoring functional vision to visually impaired individuals.

## 1. Introduction

Visual impairment is a pervasive global issue affecting millions of individuals (Bourne *et al* 2020). Those living with visual impairment experience profound and multifaceted impacts on their physical (Krumpaszky and Klauss 1996), cognitive (Zheng *et al* 2018, Lim *et al* 2020), emotional (Evans *et al* 2007), psycho-social (Salminen and Karhula 2014, Swenor *et al* 2020), and financial well-being (Köberlein *et al* 2013). Furthermore, visual impairment has substantial socioeconomic consequences (Eckert *et al* 2022, Rein *et al* 2022). As growing and aging populations make the global burden of blindness and visual impairment a pressing issue, exploring visual prosthesis devices as a treatment has shown considerable promise. With many visual prosthesis devices on the market (summarized in Makawi *et al* 2024) and others in clinical trials, this review aims to evaluate the current state of visual prostheses with regard to the perception of motion they can offer.

As of this writing, many different visual prosthesis systems have either been approved for clinical use, are currently in, or are nearing human clinical trials. These include the Alpha IMS/AMS, Argus II, CORTIVIS, EPI-RET, ICVP, IRIS, Orion, PRIMA, and others (summarized in Makawi *et al* 2024). However, while some of these systems are in active development, some have been abandoned or are no longer available; the implications for implanted patients have been discussed in the press (Strickland and Harris 2022) and scientific literature (e.g. Ramirez *et al* 2023). While much of the existing clinical work has concentrated on the retina, other labs are developing prosthetic systems to stimulate visual areas outside of the retina (reviewed in Mirochnik and Pezaris 2019), but with a few exceptions, the majority of this later work remains in pre-clinical testing.

In this review, we will summarize the current body of knowledge concerning motion perception in artificial vision and outline the importance of concentrating on this aspect of prosthesis function. Starting with a review of the neurological basis of motion perception, we will cover perception of frontoparallel motion, the most common paradigm used to assess motion processing in artificial vision. Tests of motion perception have been studied in visual prostheses that stimulate several different anatomical locations, including the retina, the visual cortex, and area MT. In addition to visual prosthesis devices, we will review the current literature on motion perception in artificial vision simulations. Finally, we will discuss the broader implications and challenges for achieving reliable motion perception in prosthetic vision. A summary of the systems considered during this review can be found in table 1, along with additional themes and concerns in table 2.

**Table 1.** Comparison of prosthesis approaches and motion perception. This table compares major classes of visual prostheses based on implantation site, mechanism of action, clinical or experimental status, and their implications for motion perception. For each approach, the table lists key advantages and limitations, representative devices or descriptive publications, estimated visual field and resolution, and whether motion perception has been studied in published work. This summary shows the range of engineering strategies currently under investigation and the varying degrees of empirical support for motion perception across approaches.

Approach	Mechanism	Advantages	Limitations	Examples	Visual field	Resolution	Motion studies
Epiretinal	Electrical stimulation of ganglion cells from the inner retinal surface	Established clinical use; direct access to output neurons	Requires intact inner retina; head-fixed camera disrupts gaze linkage	Argus II (Second Sight; discontinued)	∼20°	60 electrodes	Dorn *et al* (2013), Ho *et al* (2015)

Subretinal	Photodiode array implanted beneath the retina to stimulate bipolar cells directly	Naturalistic input by imaging external world; eye-movement integration	Requires functional bipolar cells; image quality depends on light conditions	Alpha AMS/IMS (Retina Implant AG; discontinued), PRIMA (science corp)	∼15°	Alpha AMS: 1600 microphotodiodes; PRIMA: 378 pixels	Stingl *et al* (2015), Edwards *et al* (2018)

Suprachoroidal	Electrode array placed between sclera and choroid, stimulating outer retina	Safer, less invasive surgery; good biocompatibility	Greater distance from target neurons reduces spatial resolution	Bionic vision Australia (BVA) prototype, Monash Vision Group	∼10°–30°	44–98 electrodes	Titchener *et al* (2020)

Thalamic	Stimulation of the lateral geniculate nucleus (LGN)	Applicable for patients with retinal/optic nerve damage	Less explored clinically	Research prototypes; Pezaris and Reid (2007); Phosphoenix, BV	Potentially full field	Experimental (1000–2000)	None

Cortical	Stimulation of visual cortex (typically V1 or extrastriate areas)	Applicable for patients with retinal/optic nerve damage	Poor motion selectivity; limited spatial integration	Orion (Cortigent) ICVP (IIT) CORTIVIS (UMH)	∼10°–20° (modular islands)	Orion: 60 electrodes; ICVP: 256 (modular); CORTIVIS: 100 (modular)	Beauchamp *et al* (2020)

Optogenetic	Light-sensitive proteins inserted into retinal or cortical neurons	Cell-type specificity; potentially high resolution	Experimental; requires gene therapy and light delivery systems	Nirenberg and Pandarinath (2012)	Potentially full field	Potentially high	Gauvain *et al* (2021)

Magnetic, ultrasound	Non-electrical stimulation modalities targeting visual pathways	Possibly less invasive	Early-stage research; unclear spatial/temporal resolution	Lee *et al* (2016a), Naor *et al* (2016), Lee *et al* (2016b)	Unknown	Likely low	None

**Table 2.** Comparison of prosthesis design factors and motion perception. This table summarizes key design features that influence motion perception in visual prosthesis systems. Factors include system resolution, field of view, gaze-contingent processing, motion encoding algorithms, and training or rehabilitation protocols. For each factor, the table outlines its underlying mechanism, potential advantages, and limitations, along with illustrative examples where available. The comparison highlights how engineering trade-offs and system-level choices shape perceptual outcomes in artificial vision.

Design Factor	Mechanism	Advantages	Limitations	Examples
Gaze-contingent systems	Image rendering adjusts in real time based on eye position	Supports spatial updating; improves perceptual stability	Requires eye tracking	Simulated systems (e.g. Paraskvoudi and Pezaris 2021, Rassia *et al* 2022)
Motion encoding algorithms	Artificial enhancement of motion cues	Improves motion detection in prosthetic vision	May introduce unnatural percepts; design-specific trade-offs	Brightness as depth; background subtraction; saliency filtering
Training and rehabilitation protocols	Repeated exposure and behavioral training to improve motion perception	Enhances performance over time; low cost	Requires user commitment; real-world performance varies	Orientation and motion training studies
Resolution	Limited by electrode count and spacing	Supports basic object recognition	Low resolution; limits reading and faces	Argus II (60); Orion (60); Alpha AMS (1600)
Field of view	Set by implant size and layout	Improves spatial awareness	Typically narrow (<20°); trade-offs with resolution	Argus II (20°); PRIMA (7–10°)

## 2. Background

Visual motion information originates in the retina and passes through the lateral geniculate nucleus (LGN) before reaching the cortex. The LGN serves as the primary relay station for parvocellular (P), magnocellular (M), and koniocellular (K) processing streams, each of which is specialized for different aspects of visual processing (Solomon 2021). While most motion-related data is found in the LGN’s M layers, P input plays a significant role in shaping spatial, temporal, and chromatic motion cues in various motion-specific brain areas (Nassi *et al* 2006), and K layers are known to be motion sensitive (Hendry and Reid 2000, Renshaw and Pezaris 2025).

From the LGN, M/P/K pathways project via the optic radiations to the striate cortex (V1) in the occipital lobe. V1, the first stage of integrated visual processing and a focus of visual prosthesis development (Schiller and Tehovnik 2008, Foroushani *et al* 2018), exhibits retinotopic organization and primarily responds to stimuli within small, well-defined receptive fields (Lian *et al* 2021). Emerging evidence suggests that V1, as well as other cortical visual centers (V2/3), also respond to motion, particularly interblob cells that are sensitive to object movement (Dupont *et al* 1994, Seiffert *et al* 2003).

Visual information progresses anteriorly from V1 to higher-order visual processing centers. A critical center for motion perception is the middle temporal area (V5 or MT, often considered in conjunction with V5A/MST, and stylized as MT+ or the MT complex), located on the posterior edge of the superior temporal sulcus (Born and Bradley 2005). MT+ neurons are highly sensitive to motion direction and speed but unresponsive to object characteristics (Dubner and Zeki 1971, Van Essen *et al* 1981, Lagae *et al* 1993). Bilateral MT+ lesions result in impairment of motion detection, speed determination, and direction discrimination (Schiller 1993). Nevertheless, the eventual recovery of some motion perception following bilateral MT+ lesions suggests the involvement of other cortical regions in motion processing despite the primacy of MT+ (Yamasaki and Wurtz 1991).

## 3. Important distinctions in motion perception through artificial vision

When discussing motion perception through artificial vision, it is important to make two distinctions. The first distinction concerns the difference between movement of phosphenes themselves and the perception of objects that are in motion when viewed through a collection of phosphenes. The second distinction concerns the difference between tasks that specifically assess motion perception, such as drifting grating detection, and tasks which require motion perception for correct completion but do not specifically measure it, such as eye-hand coordination tasks. We will review these two ideas in the following two sections.

### 3.1. Moving phosphenes versus seeing moving objects through phosphene vision

**Figure 1.**
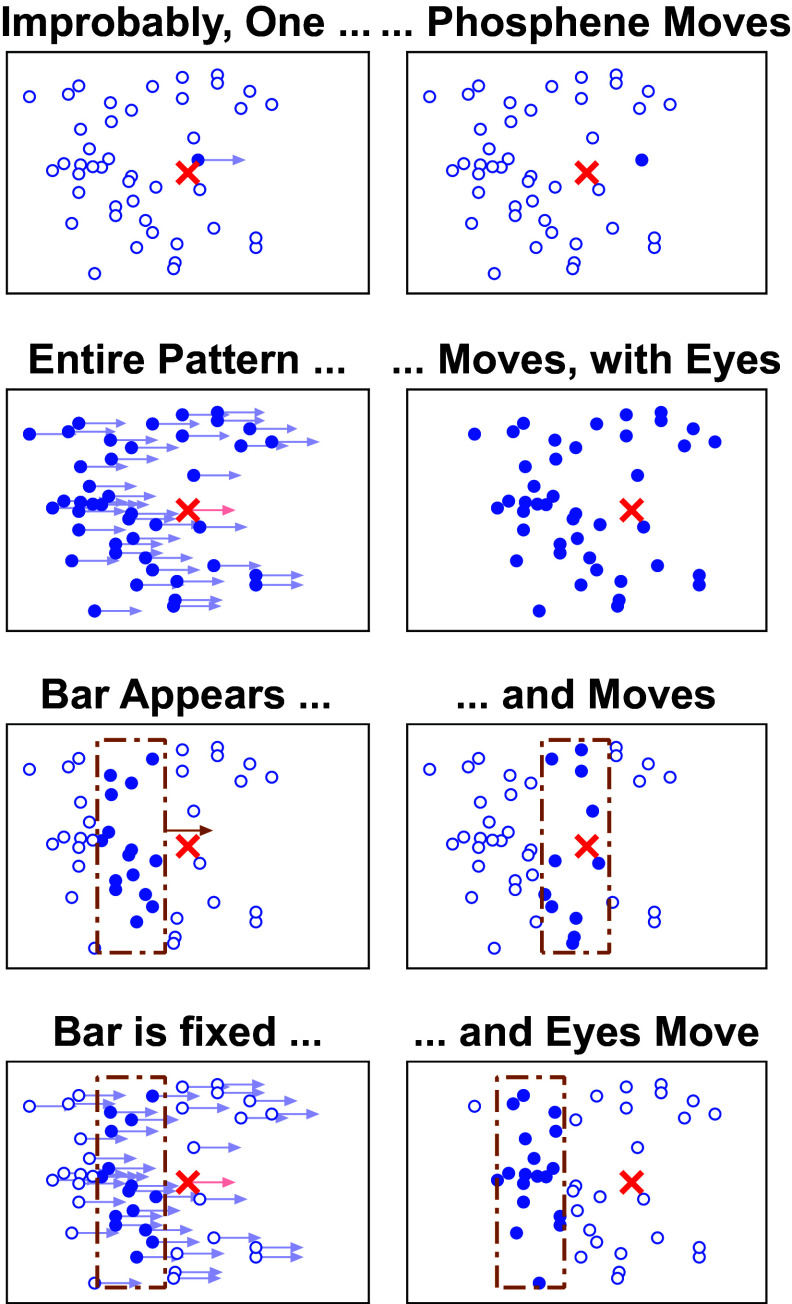
Different kinds of motion. Four types of motion are illustrated with before-and-after plots. Each circle represents the perceived location of a phosphene induced by a specific electrode in the prosthesis, relative to the gaze location (red cross); when electrodes are activated, their phosphenes are perceived (filled circles), but when not activated, their phosphenes are not seen (open circles). The top row shows an unlikely scenario where a single illuminated phosphene shifts relative to the rest while the gaze position remains fixed. The second row shows eye movement, causing the entire retinotopically-anchored phosphene field to shift. The third row depicts the gaze being fixed, and thus the phosphene field fixed as well, as a bar moves (dashed lines), changing the subset of illuminated phosphenes representing it, and creating apparent motion. The fourth row depicts the converse condition with gaze moving and the bar fixed. The apparent motion is in the opposite direction, exactly offsetting the gaze shift, so that the brain can interpret the object as stable in the world. Most experiments in this review are variants of the third row. The pattern of phosphenes used in this figure is modeled after measurements from a patient implanted with an Orion device (Oswalt *et al* 2021). See Paraskevoudi and Pezaris (2018, 2021) for additional detail regarding gaze contingency effects.

Phosphene movement is not the same as motion perception derived from phosphene vision. Phosphene movement does not inherently yield motion perception, and motion perception does not necessarily rely on phosphene movement (figure 1). In the visual system, motion and form processing are closely intertwined and can co-occur (Pavan *et al* 2013), yet one may dominate depending on how visual information is presented (Donato *et al* 2020).

Early work by Brindley and Lewin (1968) noted that electrically stimulating the primary visual cortex produced phosphenes which followed eye movements, giving the phosphene the appearance of a fixed position in retinotopic space (that is, always at the same relative position to the point of regard), as confirmed by many later reports (Dobelle and Mladejovsky 1974, Schmidt *et al* 1996, Caspi *et al* 2021) for different stages of the early visual pathway (Chapanis *et al* 1973, Veraart *et al* 2003, Pezaris and Reid 2007, Caspi *et al* 2009). Because the visual circuitry compensates for eye position in motion processing, this apparent motion in phosphenes driven by eye position changes is often ineffective at conveying motion within the visual scene unless paired with a system that incorporates eye position in its translation of the scene to phosphene stimulation (Paraskevoudi and Pezaris 2018, 2021).

However, there have also been reports of individual phosphenes that move within the retinotopic field, independent of eye position. Studies show that electrode-generated phosphenes can yield movement and form perception (Wilke *et al* 2011, Sinclair *et al* 2016); participants reported perceptions of both form (‘an arc,’ ‘a slightly curved horizontal line’) and motion (‘movement,’ ‘swept across the visual field’) when one or multiple electrodes were activated. These reports seem to be more the exception than the norm, however, and may have been related to confounds such as high levels of ongoing spontaneous phosphenes, and uncontrolled nystagmus. In contrast, form perception was evoked from line tracing of figures in recent research using current-steering to move individual phosphenes (Beauchamp *et al* 2020). Participants could identify and reproduce patterns created by moving phosphenes, demonstrating how motion alone can convey form information.

The primary means for conveying motion through a phosphene array does not require phosphenes that move, but rather a moving pattern of activation (figure 1). Most current visual prostheses successfully evoke motion perception using electrodes that are fixed in place in the tissue, producing phosphenes that are fixed in retinotopic coordinates (Ayton *et al* 2014, Chuang *et al* 2014, Lewis *et al* 2015). Like with a television, computer screen, or virtual reality headset, successive activation of pixels that themselves do not move can still create the perception of motion, often called apparent motion (Wertheimer 1912, Ramachandran and Anstis 1986). However, technical limitations, such as electrode count, make motion perception challenging in artificial vision (Picaud and Sahel 2014, Stingl *et al* 2017). For the remainder of this review, we will focus on motion perception evoked by patterned activation and discuss the movement of individual phosphenes only when it intentionally contributes to motion perception.

## 4. Frontoparallel motion perception in artificial vision

**Figure 2.**
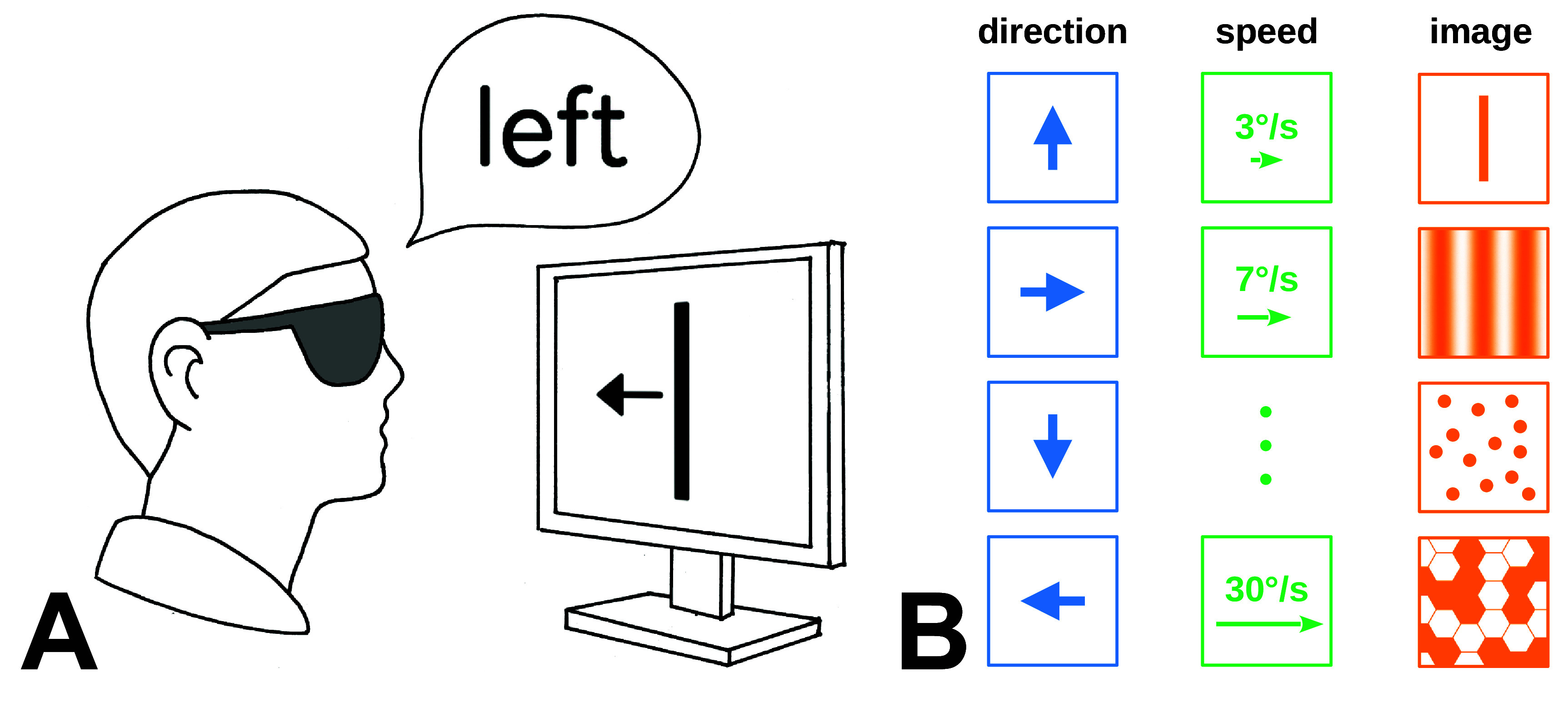
BaLM motion task. (A) The motion detection and identification task from the BaLM suite has patients using their visual prostheses to view moving images on a computer monitor and report the direction seen. Subjects respond with the direction of motion either by verbal identification, by button press, or by touch screen, depending on the study. (Drawing by Zofia Pezaris). (B) Stimuli include motion in one of four directions (first column, in blue) or eight (not shown) and at varying speeds (second column, in green). Image types ordered by complexity include bars, gratings, random dots, and patterns (third column, in orange), although only one image type is typically used in a given experiment. One variation of this test uses a continuous selection of direction, rather than a fixed set of four or eight, with subjects reporting direction via touch screen; the error angle between reported and actual directions is then analyzed. For moving dot stimuli, it should be noted that the moving dots are not themselves phosphenes, but typically an image of a texture that moves rigidly, unlike seminal experiments in MT where the dots may not all move in the same direction (e.g. Britten *et al* 1992). The motion test in the original BaLM suite used hexagonal patterns like the bottom image in the third column (after figure 1 of Bach *et al* 2010).

The most basic test of visual motion perception assesses motion detection in the frontoparallel plane (Nakamara and Otsuka 1995). Many studies examining motion perception in artificial vision use tasks similar to the basic assessment of light and motion (BaLM) (Bach *et al* 2010). The BaLM suite is a validated set of tests that quantify visual acuity in low vision. Of particular interest here is the motion task in which a random pattern of light and dark hexagons begins to move in one of four or eight directions. Subjects indicate the motion’s direction in a four- or eight-alternative forced choice task (figure 2).

There have been many variations of this task used in the literature, challenging comparability across studies. BaLM adaptations have adopted moving lines or a moving dot pattern rather than the hexagons pattern. Another common variation eliminated the alternative forced choice task, opting instead to have participants indicate the direction of motion on a touchpad (Dorn *et al* 2013, da Cruz *et al* 2016) and measuring error as the angular difference between the reported and actual directions of motion. Another variation explored different speeds within the BaLM to quantify the maximum speed at which motion could reliably be detected (Stingl *et al* 2013b, Stingl *et al* 2015).

### 4.1. Frontoparallel motion perception in retinal prostheses

#### 4.1.1. Second sight medical products: Argus I and II Retinal prostheses

The bulk of evidence for motion perception in visual prostheses comes from the Argus retinal prosthesis system, an epiretinal prosthesis developed by second sight medical instruments (Luo and da Cruz 2016). Research comes from the two versions of the implant, the Argus I, with 16 contacts, and the Argus II, with 60 contacts. Both were implanted on the inner retinal surface, designed to stimulate retinal ganglion cells (Humayun *et al* 1999) with signals from an external camera fixed in a set of goggles (Mirochnik and Pezaris 2019). The Argus II was one of the first visual prosthesis devices to obtain a CE mark (EU regulatory approval) and the only one to obtain FDA (US regulatory) approval, contributing to the preponderance of data with this device (Luo and da Cruz 2016, Farnum and Pelled 2020). Importantly, the camera, being externally worn, does not incorporate eye position into visual field movement to eye position. As naturalistic eye movements are crucial for motion perception in sighted individuals (Dodge 1904, Wallach 1982), eliminating visual field adjustments that track eye position may limit motion perception in both laboratory and daily-use settings (Caspi *et al* 2018, Paraskevoudi and Pezaris 2021, de Ruyter van Steveninck *et al* 2024).

Early Argus system research on frontoparallel motion perception had mixed results. A 2003 study using the Argus I (Humayun *et al* 2003) with one patient showed above chance identification of displacement direction of two sequentially activated phosphenes. The sequential presentation of two nearby phosphenes (within 3 s, average minimum distance about 7° of visual angle; see Humayun *et al*, figure 2(b)) might not have been expected to trigger apparent motion perception (Julesz and Payne 1968, Anstis 1970, Moleirinho *et al* 2021), so the perceptual mechanism is unclear. The participant also identified, at above chance levels, the direction of motion from the edge of a 15 cm book entering the image as it was moved up or down 5 cm from the camera; we estimate the book would have spanned the camera image and thus the array’s field of view as well. A second study, also with the Argus I (Yanai *et al* 2007), found that only one of the three participants could detect movement. The participant’s younger age and shorter duration of visual impairment suggest that other factors, such as progression in retinal degeneration, may impact restoration of motion detection ability. Both studies should be viewed cautiously due to small sample size and lack of methodological validation.

More recent research with Argus II patients used variations of the BaLM assessment, thus increasing methodological standardization and reliability. These studies utilized a direction of motion task in which a high contrast white bar moved across a black touch screen. Performance was quantified using angular response error. Many of these studies found improved motion perception with, compared to without, the device turned on (Humayun *et al* 2012, Dorn *et al* 2013, Rizzo *et al* 2014, Ho *et al* 2015, da Cruz *et al* 2016, Arevalo *et al* 2021). Although statistically significant, the practical significance of the observed improvement should also be considered. While improvement alone suggests the device’s technical capacity to generate phosphenes, evaluating absolute performance may be a better gauge of the device’s functionality as an assistive device.

Accordingly, many of these Argus II studies reported participants’ absolute performance, in addition to significance levels. Even among users experiencing substantial motion perception benefits from the device, absolute error rates remained notably high and variable. Dorn *et al* (2013) found that participant errors in identifying the direction of motion spanned from around 10° to over 100°, with 18 out of 28 participants falling within the 50°–90° range. Other studies reported mean errors across all participants to range from 55° to 65°, with variations depending on the post-implantation year (Humayun *et al* 2012, da Cruz *et al* 2016). Arevalo *et al* (2021) reported the highest average errors, measuring at 81° with the system on and 91° with it off. In Rizzo *et al*’s study (2014), the most successful participant accurately identified the travel direction on only 69% trials, however, the definition of a correct response was not specified. The reported error magnitudes, especially when using consistent and validated methodology, cast doubt on these device’s capability of providing motion perception that would be found useful by subjects (Karadima *et al* 2023). Considering the overarching objective of enhancing the quality of life for visually impaired individuals, motion misperception of these scales raise substantial safety concerns, independent of other limitations, such as narrow field of view, and would likely lead to only marginal improvements in mobility and independence.

Other studies failed to find any significant motion perception benefit using the Argus II device. Studies by Schaffraft *et al* (2019), Delyfer *et al* (2021), Yoon *et al* (2021), and Yue *et al* (2015), also using variations on the BaLM methodology, found no significant improvement in the ability to identify direction of motion using the Argus II device. When reported, absolute performance for these studies was consistent with absolute performance in studies reporting motion perception benefits. In Schaffraft *et al* and Delyfer *et al*, the average response errors were between 60°–70° at each time point (3, 6, 12, 24 months post-implantation).

The discrepancy between studies reporting motion perception benefits with the Argus II and those finding no significant improvement likely arises from differences in experimental design and levels of subject experience. Studies showing benefits (Dorn *et al* 2013, Rizzo *et al* 2014, da Cruz *et al* 2016) often used simplified or repetitive stimuli, extended presentation duration, or restricted directional choices, increasing the likelihood of above-chance performance through compensatory strategy rather than veridical perception. In contrast, studies reporting null results (Yue *et al* 2015, Schaffrath *et al* 2019, Delyfer *et al* 2021, Yoon *et al* 2021) frequently employed more naturalistic stimuli, shorter exposures, or more rigorous trial randomization, placing greater perceptual demands on subjects. Subject-level factors also likely contributed: individuals who received targeted motion training or reported regular device use were observed to perform better on these tasks (Dorn *et al* 2013, da Cruz *et al* 2016), and were hypothesized to have developed compensatory strategies not present in less experienced users (Rizzo *et al* 2014).

**Figure 3.**
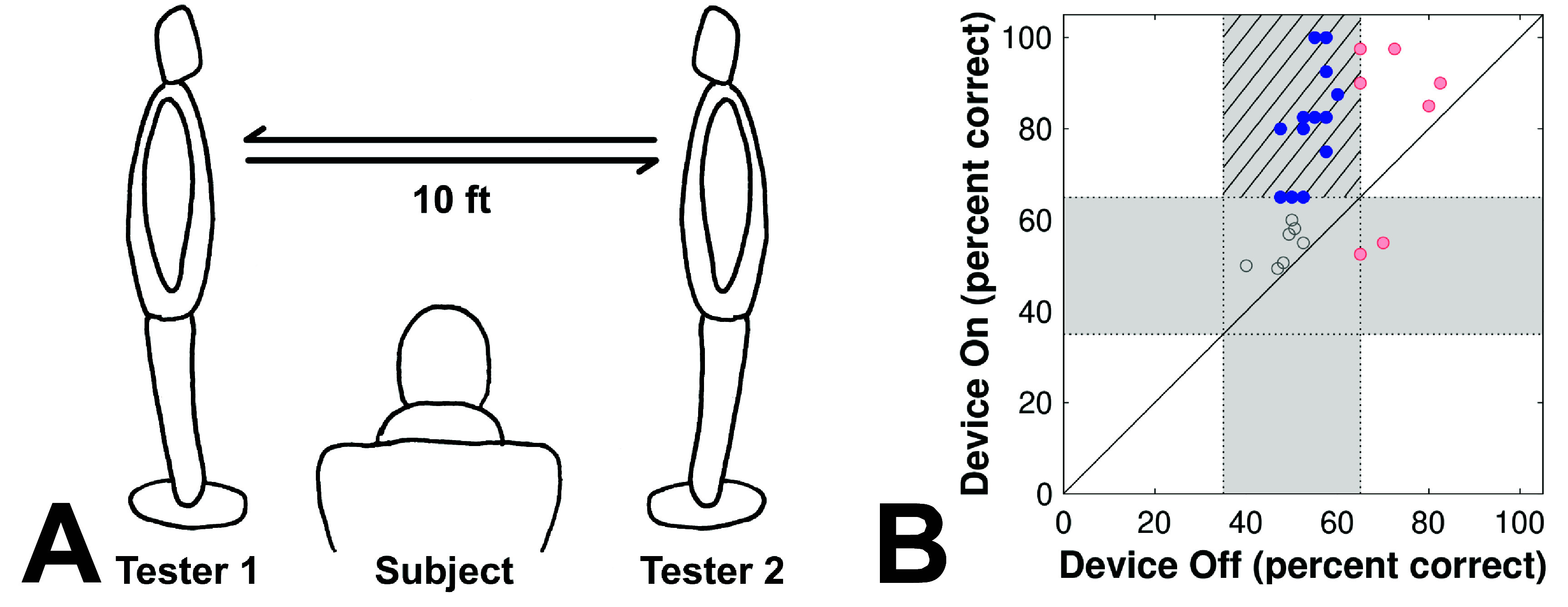
Walking motion perception test. One of the non-BaLM tests of motion perception was designed to more closely mimic a real-world experience of detecting people walking. (A) Two testers stood 10 feet apart and 10 feet in front of a subject. At the sound of a tone, one or the other tester walked to the opposite position, and, afterward, the subject reported the perceived direction of motion. (B) Results were mixed; while 25/27 subjects improved on the two-alternative forced choice test (points above the identity diagonal), a minority (13/27, filled blue) improved from chance performance (grey zones) without the device, to statistically above chance with the device (hatched zone); the remainder were already above chance without the device (unfilled red), or had no significant improvement (unfilled gray). (A, image drawn by Zofia Pezaris, after figure 4 of Dagnelie *et al* 2017; B, data extracted from their figure 9 and replotted).

Studies with the Argus II using non-BaLM methodology also find error-prone motion perception performance. In a study by Dagnelie *et al* (2017), participants indicated the direction of motion of an experimenter walking in front of them (figure 3). While external auditory and tactile cues were reduced, no sensory masking devices were used. With the device engaged, 67% of participants (18/27) indicated the direction of motion with significantly above-chance accuracy. However, of those 18, five also had above-chance performance with the device disengaged. Thus only 48% (13/27) went from non-signficant to significant performance with the device. Even with this 48%, the results should be accepted cautiously given the potential contribution of non-visual, specifically audio, motion information. While there appears to be a strong trend in improvement with the device turned on (25/27 improved), the results were inconsistent across individuals (figure 3(B)).

Overall, the Argus II appears to give rise to increased motion perception. However, this technological success must be carefully contextualized with respect to functional improvements it can offer individuals with visual impairment. The magnitude of motion perception error conferred by Argus II remains too high to support reliable and accurate motion discrimination under realistic visual conditions. Future development and research should prioritize this functional goal. In particular, the integration of eye position tracking with the external camera may be an important feature to add for improving Argus II motion perception, along with the more obvious increases in resolution and field of view.

#### 4.1.2. Retina Implant AG: Alpha IMS and AMS subretinal visual prostheses

Alpha IMS and its successor, Alpha AMS, are two other visual prostheses which have been the focus of research regarding frontoparallel motion detection and assessment. They are subretinal devices developed by Retina Implant AG (Stingl *et al* 2013b) with integrated imaging and stimulation circuitry placed between the outer layer of the retina and the pigment epithelium to stimulate intermediate layers of the retina, primarily bipolar cells. The benefit of integrating the imaging and stimulation circuitry so that both are fixed to the retina is that an external camera is not needed, as light entering the eye is converted directly to electrical impulses. Thus, users can continue to rely on natural eye movements and physiological retinal processing to steer the field of view, and the device inherently incorporates gaze contingency (Pezaris and Eskandar 2009, Mirochnik and Pezaris 2019). For motion perception, this is important perceptually, as eye movement and motion perception are highly interrelated (e.g. smooth pursuit eye movements for dynamic object tracking, or attention-related saccade eye movements at the onset of motion) (Dodge 1904, Wallach 1982). The Alpha IMS gained CE approval in 2013, while the second-generation Alpha AMS gained CE approval in 2016 (Perriello 2016).

Results from studies on first-generation Alpha IMS’s frontoparallel motion detection are mixed. Most of the studies used variations of the BaLM motion task. However, unlike Argus II studies, which calculated response error in degrees, these studies used two- and four-alternative forced choice designs, making it more difficult to assess and compare absolute performance to the Argus II.

The most promising results come from Stingl *et al* (2013b), (2013c), (2015), who used a four-alternative forced BaLM motion task with increasing speeds (starting at 3° s^−1^). They recorded the highest speed at which a participant could identify movement of a random array of dots (chance at 25% correct, passing at 62.5% or better (Stingl *et al* 2013a). In successive studies with the Alpha IMS, researchers found that 21% (Stingl *et al* 2015) and 56% (Stingl *et al* 2013a) of participants were able to accurately identify motion at any speed. Of successful participants, most showed the maximum speed for accurate direction identification between 3° and 7° s^−1^ (about 5 and 2 s, respectively, to traverse the array diagonally) with one exceptional participant able to accurately identify motion at 35° s^−1^ (about 0.4 s).

Other studies by Stingl *et al* provided important data concerning implant location on motion perception, finding that implant placement has significant influence on the patient’s ability to detect motion. A study with 20 participants 2013c) supported foveal placement for the implant for better motion detection, with 75% of participants in the cohort with foveal placement (6/8) able to accurately detect motion at the lowest speed (3° s^−1^) as compared to the cohort with parafoveal placement who could not detect motion at any speed. The authors in that report suggest that the amount of retinal remodeling, expected to be greater with increasing eccentricity for their patient group, may explain the difference in task ability between the two groups.

The Alpha AMS device improves on its Alpha IMS predecessor through changes in device material aimed at improving prosthesis lifetime, and a slight increase in electrode count from 1500 to 1600 (Daschner *et al* 2018). Despite these updates, motion detection results using the Alpha AMS and Alpha IMS are roughly equivalent. In 2017, Stingl *et al* published an interim report on Alpha AMS performance results (2017). Using the same BaLM-based methodology as their previous experiments (Stingl *et al* 2013b, 2013c, 2015), results showed that motion detection was only possible for two out of 11 patients. In 2018, another study by Edwards *et al* (2018) used the same motion detection procedures and found that none of the five participants passed the motion detection task despite subfoveal placement. The disparities in motion detection among these studies have not been fully explained but may relate to the level of retinal rewiring as mentioned above.

Like the Argus II, motion perception results from the Alpha IMS and AMS prostheses remain mixed. The introduction of different speeds in the BaLM test revealed a dependence of motion perception on speed of the motion—an important point for better understanding the real-world functional capacity of these devices. However, the use of forced-choice methodology (motion detected or not detected) as opposed to absolute response error (angular difference between stimulus and report) in these studies makes it hard to compare performance between the Argus II and Alpha AMS/IMS devices. Furthermore, performance results as reported in these studies make it hard to draw concrete conclusions about real-world functional performance. Finally, none of these studies measured nor controlled for eye movement, likely giving an advantage to the subretinal Alpha IMS/AMS implants. Future research on eye position and movement during prosthetic vision motion perception will not only help to improve motion perception in subretinal implants but may better inform how non-subretinal devices might use eye position tracking (Caspi *et al* 2018) to deliver more naturalistic prosthetic vision (Paraskevoudi and Pezaris 2021, de Ruyter van Steveninck *et al* 2024).

#### 4.1.3. Bionic vision technologies: bionic eye system suprachoroidal visual prosthesis

The bionic eye system is a third retinal prosthesis with published reports of motion detection and perception. The bionic eye system is a suprachoroidal visual prosthesis developed by bionic vision technologies (Ayton *et al* 2014). The suprachoroidal approach, as opposed to subretinal and epiretinal approaches, has advantages in ease of surgical access (Mirochnik and Pezaris 2019), as the implant is placed between the choroid and the sclera (Villalobos *et al* 2013), thus taking advantage of the remaining retinal processing that has not been lost to degeneration. Like epiretinal approaches, this device uses an external camera in order to process the visual scene (Mirochnik and Pezaris 2019), but does not include measurement of eye position to compensate phosphene delivery for gaze direction (Pezaris and Eskandar 2009, Paraskevoudi and Pezaris 2021). Currently, this project has not yet received regulatory approval.

Studies with the suprachoroidal implants have been methodologically consistent, relying on the validated BaLM paradigms. This consistency may be attributable to the recency of this research compared to other visual prostheses. The first study on motion perception through a suprachoroidal implant was by Fujikado *et al* (2011) in 2011, where two participants viewed a white box moving across their visual field either horizontally or vertically. One participant scored significantly higher than chance; the other did not. Another study by Shivdasani *et al* (2017) in 2017 used similar methods, testing motion perception of a fixed white bar in 8 directions, with 5 randomized speeds. Again, out of the two participants, only one passed the 8-alternative forced choice design, scoring above the 63% passing criterion at 4 out of 5 speeds. The fastest speed of 80 degrees/sec was the only speed at which the more successful participant failed to meet the passing criterion. The second participant was unable to perform at a passing level at any speed, even when the task was simplified to a 4-alternative forced choice paradigm. But they still performed above chance at the slowest speed, accurately resolving motion in 27% or 50% of trials depending on electrical stimulus rate (50 and 400 pulses per second, respectively). With a cohort of four participants, Petoe *et al* (2021) found similar results: just 2 of 4 participants performed significantly better with the device on (63% and 83% accurate) versus off (28% and 25% accurate) using a 4-alternative forced choice task BaLM task.

Titchener *et al* (2020) explored the influence of retinotopic map accuracy in motion discrimination when using suprachoroidal devices. Participants were able to perceive the direction of motion of a drifting bar significantly and substantially above chance in normal prosthesis operation (contrast with the drifting grating stimuli used later), with performance deteriorating to near-chance levels with scrambled phosphene location maps, demonstrating the subjects’ use of retinotopic information. Of the three speeds tested (7, 15, 30° s^−1^), subjects performed better at the lower two. The three subjects exhibited smooth-pursuit-like gaze movements consistent with stimulus direction and speed, as is associated with natural visual perception of motion. However, optokinetic-like eye movements evoked when viewing drifting gratings were highly inconsistent with stimulus motion. The combined findings suggest there was only partial activation of normal motion-processing pathways. The requirement that subjects hold their eyes fixed for normal operation, because of a lack of gaze contingency, complicates interpretation of the results: What was viewed as smooth pursuit eye movements may have been the tracking of phosphenes themselves, rather than the motion of the stimuli.

In summary, the results from the suprachoroidal implant align with those from the Alpha IMS and AMS studies, highlighting the importance of speed in motion perception. However, an interesting observation can be drawn comparing the results from the Alpha IMS/AMS systems to the suprachoroidal devices: Alpha IMS/AMS systems have inherent gaze contingency whereas the suprachoroidal systems do not. Given our understanding of how natural eye movements influence motion perception (as discussed earlier), one might anticipate better performance with the Alpha IMS/AMS devices. However, since gaze position was measured in the suprachoroidal studies but not incorporated into the device operation, and was neither measured nor controlled in the Alpha IMS/AMS studies, interpreting the results in terms of the influence of eye position and movement is challenging. Plausible explanations for the observed similarity is that suprachoroidal participants were instructed to center their gaze and hold it forward, or they may have compensated for the lack of gaze contingency by relying on head position. A study by Titchener *et al* (2020) identified two participants who exhibited head movements congruent with stimulus motion, suggesting that they may have used external cues, such as head position, to resolve motion.

### 4.2. Frontoparallel motion perception in cortical prostheses

Cortical prostheses, as opposed to retinal prostheses, stimulate the primary visual cortex to generate phosphenes. Fewer studies have explored motion perception in cortical prostheses than for retinal prostheses. However, the ideas that stimulating the visual cortex could elicit functional phosphenes (Le Roy 1755, Brindley and Lewin 1968, Volta 1997) or that the primary visual cortex is retinotopically mapped (Hubel and Wiesel 1959) are not new. A few important figures in artificial vision have focused their efforts on the development of a cortical prosthesis, including Brindley and Lewin (1968), and Dobelle and Mladejovsky (1974), Dobelle *et al* 1974, 1976, Dobelle 2000). Since then, other researchers have examined cortical prostheses’ effectiveness in generating motion perception. This review will present two published studies which examine motion detection with cortical devices.

One study by Chen *et al* (2020) measured motion perception using BaLM-inspired methodology in cortical prostheses. In this study, monkeys were trained to report the direction of motion in sequentially activated dots on a screen. Once the direction of motion task had been learned in natural vision, the stimuli were then presented in phosphene vision. All the monkeys performed significantly above chance in determining direction of motion in phosphene vision, indicating motion perception as well as direction discrimination that was comparable to control conditions with simulated phosphenes viewed through normal vision. It is important to note that the animals maintained central fixation during stimulation in this task, so that eye position was not a confounding factor.

**Figure 4.**
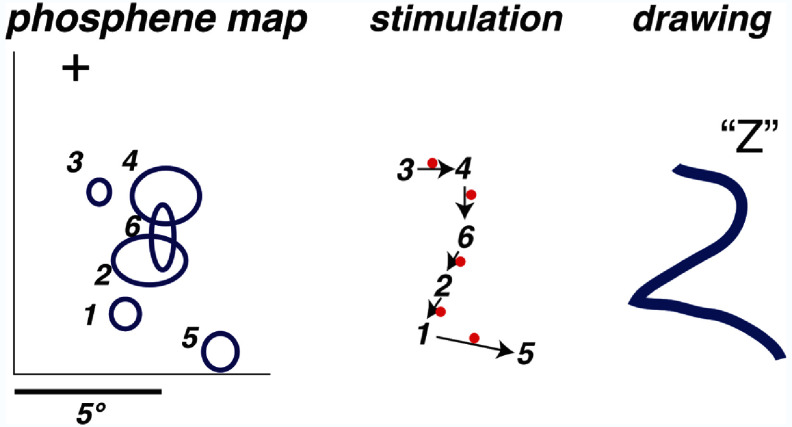
Reproduction of phosphene tracing from cortical stimulation via current steering. Left: retinotopic receptive field maps (blue ovals) for a series of stimulation sites in visual cortex with the order of stimulation marked. Center: schematic depiction of example stimulation sequence across regular (numbers) and virtual (red dots) electrodes. Right: drawing made in response to apparent phosphene motion, showing a trajectory that was recognized by the subject as the letter *Z,* demonstrating that current steering can convey both form and motion information. A similar test of sequentially activated phosphenes, but often with just two, was used in other studies as well (e.g. Humayun *et al* 2003, Yanai *et al* 2007, Chen *et al* 2020). Reprinted from Beauchamp *et al* (2020), Copyright (2020), with permission from Elsevier.

The second motion perception study with a cortical prosthesis was conducted by Beauchamp *et al* (2020). They studied how activating a sequence of phosphenes, including with current steering, a technique for increasing the placement resolution of individual phosphenes without the potential complications from increased electrode counts (Meikle *et al* 2022, Meikle and Wong 2022), can elicit form and motion perception (figure 4). Current steering has been used in retinal (Dumm *et al* 2014, Spencer *et al* 2018), optic nerve (Yan *et al* 2016), and cochlear (Firszt *et al* 2007, Landsberger and Srinivasan 2009) implants, among others. In their experiment, current steering was employed to generate the movement of a perceived phosphene, where the location of a virtual electrode between two physical electrodes was modulated by changing the relative charge at each physical electrode (Meikle *et al* 2022). The resulting phosphene moved along a specific path, providing form information (i.e. drawing letters) and motion information (i.e. direction of motion). The results showed that the participant performed well above chance, indicating the correct direction of motion 87% of the time (chance was 50%), and recognizing the drawn letter 66% of the time (chance was 25%). Notably, in this study eye position was neither measured nor controlled, although the subjects were instructed to help keep their eyes fixed in space by placing a finger on the monitor in front of them.

The results of motion perception studies in cortical prostheses are promising but limited, with few publications, low sample sizes, and a lack of consistent methodology. To determine the potential for functional motion perception in these devices, more experiments using standardized methods are needed. To maintain consistency and validity, standards such as the BaLM suite developed for use in retinal prostheses (Bach *et al* 2010) should be applied. Furthermore, as with retinal prostheses, gaze position compensation is an integral requirement to establish motion perception that rises to substantial fractions of normal visual ability. Since phosphenes from cortical stimulation are retinotopically mapped, if eye position is not used by a cortical prosthesis to correct the camera view, phosphenes (always in retinotopic coordinates) will appear to move along with the eyes, confounding motion perception in real-world use.

## 5. Augmented representation of motion in visual prostheses

We have seen that objectively measuring motion perception in visual prostheses can be challenging, with often unsatisfactory results. One potential solution is to use technology from machine vision, which uses machine learning to extract, analyze, and interpret visual information (Wang *et al* 2021); with this additional scene analysis, a visual prosthesis might distill camera output in order to enhance the presentation to subjects in specific ways. Although none of the studies reviewed thus far tested the use of machine-vision techniques to specifically enhance motion perception, related filters are already in some visual prosthesis devices, such as color processing, edge detection, and contrast inversion in the Argus II (Gilhooley and Acheson 2017) or image processing algorithms in the IRIS device (not discussed in this review) which mimic retinal temporal resolution (Fauvel and Chalk 2022).

How to best analyze and translate raw scene data for machine-vision enhanced visual prostheses, the *encoding problem*, remains an open question (reviewed in Wang *et al* 2021). Of the attempts to use machine vision methods specifically for motion perception in visual prostheses, the majority of studies have focused on navigation tasks. As other papers have done comprehensive reviews on machine vision in visual prostheses (Barnes 2012, Perez-Yus *et al* 2017, Wang *et al* 2021, de Ruyter van Steveninck *et al* 2022), we will concentrate on motion perception alone.

Two common scene processing strategies from machine vision with application to visual prostheses are *intensity filtering* and *depth filtering*. Intensity-based visual presentation, or *veridical encoding,* is how information is classically presented in visual prostheses; objects which emit more light to the camera are represented through brighter phosphenes. Depth filtering uses brightness to encode closeness to the camera; objects that are closer in the scene appear brighter in phosphene vision. Thus, objects moving toward a user, which are a potential threat, become brighter as well as larger, whereas objects moving away from a user become dimmer as well as smaller. Although depth filtering fundamentally enhances position, rather than motion directly, the change in position (and representative change using depth filtering) may be useful in detecting looming motion, for example.

Research examining the impact of depth filtering on motion perception in visual prostheses has shown that depth filtering is effective in obstacle avoidance tasks, especially in environments with hanging obstacles, and outperforms intensity filtering in some cases (Barnes *et al* 2011, Lieby *et al* 2011). In a 2020 study (Kartha *et al* 2020) employing the Argus II, the incorporation of depth filtering markedly enhanced motion direction detection in a task assessing the direction of motion of individuals on an escalator from 5–6 m away. Detection rates were 6%–21% (depending on the participant) without depth filtering and increased to between 69% and 73% with depth filtering. Other studies have used more advanced depth filtering algorithms, such as visually differentiating between the ground and other non-ground surfaces, which further improves obstacle avoidance performance (McCarthy *et al* 2014).

Another possible technique is *background subtraction*. Background subtraction is similar to depth filtering. In this paradigm, rather than depth being presented as a gradient of brightness, objects in the foreground are isolated from the background and presented alone. One study of sighted subjects viewing a simulation of artificial vision found that background subtraction of dynamic stimuli in a motion detection task significantly improved performance from about 50% to above 75% correct (Wang *et al* 2014). This type of filtering is useful for perception of individual moving objects (such as frontoparallel motion) or other cases where camera location remains fixed, however, it may impede performance in navigation or other self-motion paradigms.

A more complicated image filtering technique called *saliency filtering*, uses a combination of intensity, depth, saturation, and edge detection to brighten the most salient or prominent visual images. Combined, these factors highlight important information in the scene better than depth filtering alone can. Studies examining saliency filtering have found that these representations appear subjectively more useful to sighted viewers (Mccarthy *et al* 2013) and can have marked benefit to obstacle avoidance in new environments (Parikh *et al* 2013).

Other machine vision techniques aimed at improving motion perception in visual prostheses are *free space filtering* and *time-to-contact* representation. Free space filtering highlights free, traversable space for the user based on information about their movement, orientation, and objects in the scene, and is well-suited for navigation tasks (Perez-Yus *et al* 2017). Time-to-contact representation, on the other hand, uses a ratio of relative velocity and distance to determine object brightness, allowing for better threat detection by making faster or closer moving objects brighter (McCarthy and Barnes 2012). While this method has broader motion perception benefits, it may not effectively represent frontoparallel motion, as objects moving orthogonal to the observer are not more noticeable than stationary objects at the same distance. These two techniques have not been tested in clinical settings.

Thermal imaging, when incorporated into visual prostheses, has shown considerable promise for enhancing motion perception (Sadeghi *et al* 2021) and supporting mobility (He *et al* 2020, Sadeghi *et al* 2024) in artificial vision. Specifically, thermal sensors provide a signal that highlights warm objects—such as humans, animals, or vehicles—even in low-light or visually complex environments, thereby improving salience and aiding in object tracking. For instance, Sadeghi *et al* (2021) demonstrated that integrating thermal imagery allowed prosthesis users to better detect and follow people (warm objects) walking in building interiors (cooler backgrounds). These findings suggest that thermal imaging and perhaps other spectral enhancements may be valuable additions to artificial vision systems and should be further explored for their potential to improve functional outcomes in real-world settings.

## 6. Discussion

The results surveyed in this review underscore a striking variability in motion perception performance across individuals and task designs, which has important implications for future visual prosthesis development. While some participants exhibit marked capabilities with certain devices or stimulus conditions, others show little or no perceptual benefit, even under similar experimental protocols. This inconsistency highlights shortcomings in our knowledge of how device characteristics, placement variability, and residual visual pathway integrity interact to provide interpretable input to the dorsal stream and, critically, what characteristics of that input make it interpretable. We encourage continued adoption of the BaLM suite to standardize motion perception assessments while also recognizing that an expansion of that suite to functionally relevant benchmarks that incorporate wider contextual frameworks is necessary.

The variability of results also echoes the complexity inherent in replicating natural motion perception through artificial vision. In the discussion that follows, we explore the implications of these findings, emphasizing the importance of standardized and contextually meaningful assessment measures, the integration of gaze position tracking, and emerging technological developments that hold promise for substantially enhancing prosthetic vision. Additionally, we propose experimental directions aimed at deepening our understanding of the neural mechanisms underlying artificial motion perception and improving functional outcomes for prosthesis users.

### 6.1. Measurements and reporting

There are two distinct measures by which one can assess quantitative success in motion perception for visual prostheses in laboratory settings: statistical significance and absolute performance. While each of these measures have strengths and weaknesses, they are fundamentally different. Each of these two measures will be considered here, bearing in mind that a well-designed experiment will create a homology between statistical significance and a generalizable real-world outcome.

#### 6.1.1. Statistical significance

Clinical studies have specific criteria for success that often include statistically significant improvement in some measurement. But from a scientific standpoint, it is important to consider whether measured differences are substantial as well as significant: do the results convey or demonstrate an understanding of a phenomenon or mechanism? For example, individuals with retinitis pigmentosa (RP, a common blinding disease) can have very poor motion perception compared to normally-sighted individuals and profoundly blind individuals have none (Turano and Wang 1992). A hypothetical improvement in motion perception from blindness to the level of RP function can be significant, without being useful. It is important to examine both the significance of measured differences as well as their magnitude in comparison to normal vision to understand the potential implications for device utility, as prospective users concentrate on functional ability, such as recognizing individuals or detecting objects for safe mobility, not statistical significance (Keeffe *et al* 2010, Lane *et al* 2011, 2012, Karadima *et al* 2023)

#### 6.1.2. Absolute performance and contextualization

Across the studies presented here, there was limited agreement on absolute performance. Performance ranged from 6% to 100% based on the specific task or individual, with only a few studies providing explicit thresholds for success. Some studies utilized chance level or forced choice tasks as a method of determining success. Although the forced choice paradigm is a well-validated tool for assessing absolute performance and provides a specific success threshold (Zavala 1965, Burgess 1995, Jäkel and Wichmann 2006, Stingl *et al* 2013a), those success thresholds should be contextualized by their functional relevance. For example, visually assessing the speed of a baseball with 75% accuracy might translate to a successful career in sports media, while visually assessing the relative speed of an incoming car with the same 75% accuracy might result in a fatal outcome. Accordingly, examining raw absolute performance data alone in the absence of any contextualization makes it hard to describe to prospective users what tasks they will and will not be able to do with a given device. Nuance and detail is necessary as long as artificial vision remains less capable than normal vision.

### 6.2. Gaze contingency

The integration of gaze position in visual prosthesis either inherently in the design, or by addition of gaze tracking, will improve motion perception in visual prosthesis (Caspi *et al* 2018, Paraskevoudi and Pezaris 2018, 2021, de Ruyter van Steveninck *et al* 2024). Many aspects of motion perception, including tracking moving objects as well as creating a spatial representation of movement (Thomas and Lleras 2007), are linked to eye movements. Therefore, visual prostheses which can incorporate natural eye movements have a greater chance of providing useful motion perception to the user.

**Figure 5.**
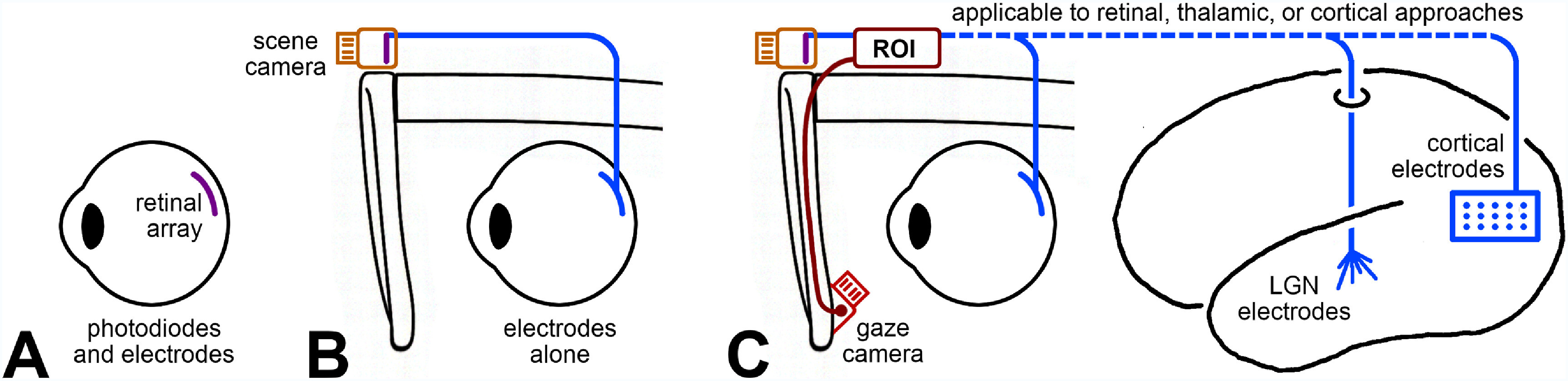
Functional configurations of visual prosthesis systems with respect to gaze compensation. (A) Subretinal prostheses with integrated photodiodes and stimulating electrodes preserve gaze contingency naturally: the retinal image moves with the eye, maintaining world-referenced stability of phosphenes once the brain applies the efference copy of eye position to retinal output through the visual pathway. (B) Epiretinal prostheses using a fixed scene camera lack gaze information, so the retinal image remains fixed while the eye moves, causing misalignment between gaze and phosphene position. (C) Systems that incorporate an eye-tracking camera and active compensation can restore alignment by selecting a region of interest (ROI) from the camera output based on gaze direction and modifying stimulation delivered to the electrode array to be centered on that area. Any approach enhanced in this way will provide signals to the brain that more accurately reflect the visual changes normally produced by voluntary movement of the eyes, thereby improving device usability.

Visual prosthesis systems differ markedly in whether and how they account for eye movements (figure 5). In the first class of devices, including many subretinal implants, the imaging and stimulating surfaces are combined and fixed to the retina and thus move naturally with the eye so they automatically compensate for eye movements (figure 5(A)). These devices require no patient training on how to control gaze, as the prosthetic interface closely replicates the function of eye movements. In the second class, systems use an external scene camera that lacks any means of adjusting the camera field of view to track the gaze position (figure 5(B)), breaking the alignment between visual percepts and world coordinates. Devices without gaze compensation require the patient to suppress eye movements, fixing their eyes in an unnatural, straight-ahead position, and scan the scene with head movements alone, with significant, negative impacts on user experience and outcomes (Erickson-Davis and Korzybska 2021). In the third class, an eye-tracking camera is incorporated to shift a region of interest (ROI) from the camera image, so that phosphene activity matches the gaze view, providing a stable presentation of the external world (figure 5(C)). Although not yet widely implemented, this architecture can be used with retinal, optic-nerve, thalamic, or cortical stimulating arrays to create gaze-contingent artificial vision. Each configuration fundamentally shapes perceptual experience, particularly for motion processing, and help clarify why seemingly similar devices can lead to divergent functional outcomes.

However, even with more phosphenes, better machine vision algorithms, or more refined biotechnological approaches for implantation and stimulation, the study of motion perception in visual prostheses will remain impeded by an inability to effectively assess and compare performance. This diminishment directly results from a lack of methodological consistency which is especially apparent in current real-world motion perception tasks. Tasks should be methodologically valid and contextualized so that performance can be compared across tasks and devices and, importantly, understood in comparison to normal vision. A given hypothetical device will likely provide a wide range of absolute performance depending on the specific task (e.g. a direction-discrimination versus a motion-speed-threshold task). This variation may be attributable to the amount or type of motion perception required for task performance, or the specific presentation of that motion. Thus, a battery of tasks may prove the most useful, not only to provide fundamental insight into the perception of motion through artificial vision and the mechanisms of engagement of higher-order visual areas, but also to assess a specific device’s utility across several different scenarios. It remains paramount that individual methodologies be rigorously motivated, validated, quantified, and, where possible, standardized.

### 6.3. Emerging technologies

Near-future advances in visual prosthesis development hold substantial promise for improving motion perception by addressing current technical limitations. One example is PRIMA (Palanker *et al* 2020), a very high resolution subretinal device implanted in perifoveal areas of geographic atrophy in macular degeneration; with integrated imaging and stimulation circuitry, it naturally incorporates eye movements. While outcomes have not yet been reported, it has the potential for excellent motion perception through a design that fuses artificial vision with remaining peripheral natural vision (Palanker *et al* 2022). Similarly, the Phosphoenix thalamic prosthesis, currently in pre-clinical development, targets the LGN with greater precision and resolution than the devices discussed above. Across all devices, increasing electrode density and improving spatiotemporal encoding are key to refining motion cues, especially for fast or complex trajectories. Coupled with improvements in gaze tracking and advanced vision algorithms, these innovations may enable prosthetic systems to support functionally meaningful motion perception. Still, assuming that better technical capacity alone will provide better performance may be misguided; the results we have reviewed above suggest that fundamental gaps remain in our understanding of the basic science of perception through artificial vision.

### 6.4. Proposed experiments

**Figure 6.**
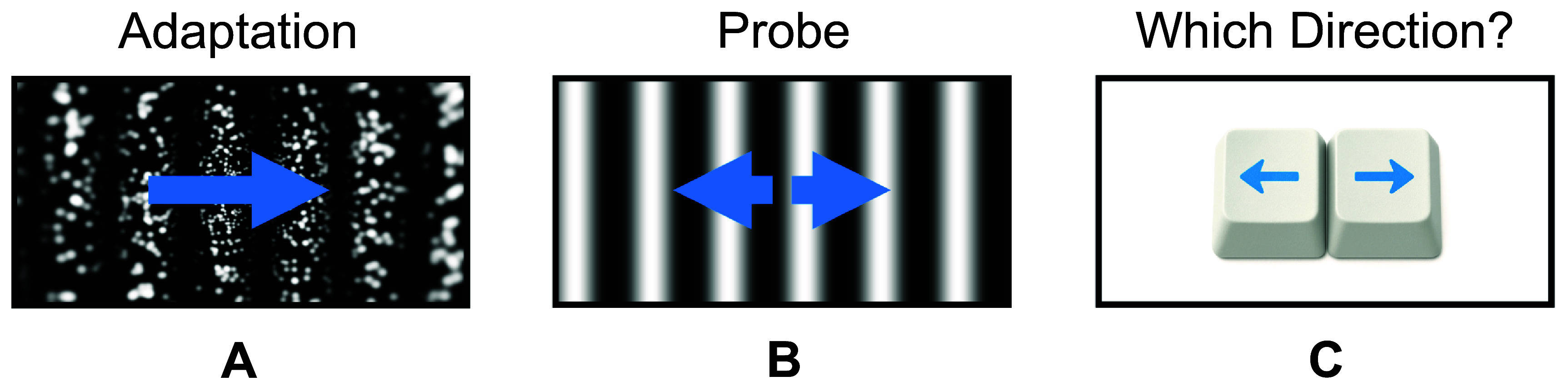
Motion aftereffect task. This task is designed to elicit a motion aftereffect (MAE) by presenting a prolonged adaptating stimulus followed by a perceptual probe. (A) Participants view a drifting grating (here shown through phosphene vision) for an extended period, holding their gaze fixed. This first adapting stimulus habituates motion circuitry in the MT/MST complex that, like afterimages, can influence the perception of subsequent stimuli. (B) After the adapting stimulus, participants briefly view a probe stimulus (here shown in normal vision) and (C) report its apparent motion, for example through a keypress. Probe stimuli typically drift at lower speeds than adapting stimuli (indicated by shorter arrows), and could also be static. Any shift of the psychometric curve fitted to probe responses versus the actual probe speed and direction reflects the depth of habituation within the MT/MST complex, and thus the level of perception of motion afforded by artificial vision.

**Figure 7.**
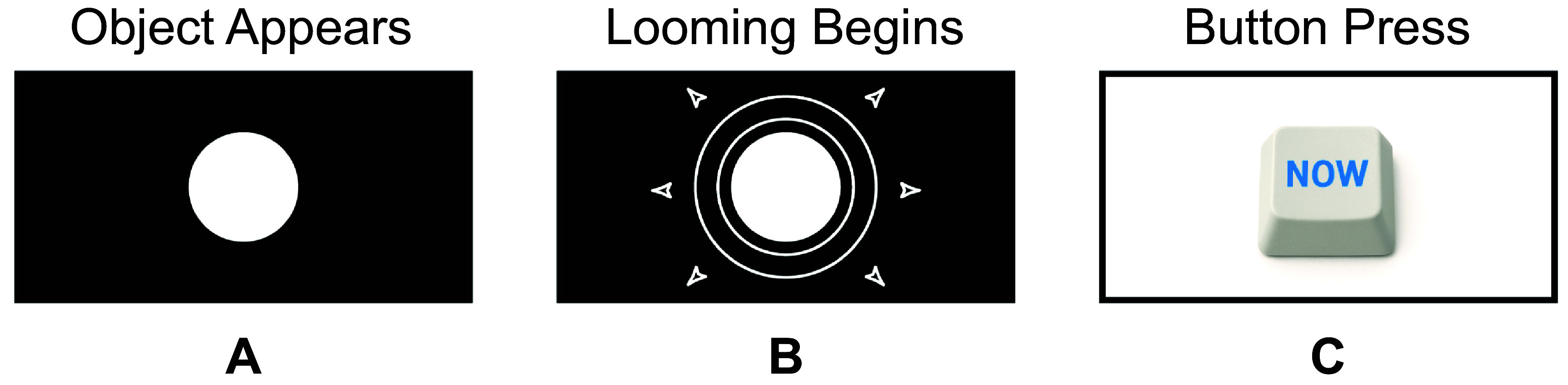
Looming motion discrimination task. This task is designed to measure the ability to discern motion of an object toward or away from the viewer. (A) Participants are shown a static object (here in normal vision. (B) After a variable amount of time, the object begins to change size, simulating an object that has started looming toward or away from the observer. (C) Participants indicate when the object appeared to approach or recede via a button press. The task probes motion-in-depth perception, and can be used under conditions designed to emulate dynamic changes in distance or size during motion-in-depth.

Based on the findings of this review, we propose two lines of inquiry to further the understanding of motion perception through visual prostheses. The first is to develop our knowledge of how artificial vision activates the dorsal stream. We propose exploiting the motion aftereffect, a perceptual illusion in which prolonged viewing of motion in one direction causes a subsequent stationary pattern to appear to drift in the opposite direction, reflecting adaptation in motion-sensitive neurons of the MT+ complex (Tootell *et al* 1995, He *et al* 1998, Huk *et al* 2001). By examining the depth of effect, we can assess the perceptual strength of motion relative to normal vision. This task (figure 6) is amenable to presentation in implanted devices, simulations of artificial vision with normally-sighted subjects as depicted in the figure, and pre-clinical animal experiments. The second line of inquiry is to examine non-frontoparallel motion by investigating looming perception, the ability to detect and interpret approaching objects as their visual size increases, also expected to engage MT+. By examining perceptual thresholds for looming (figure 7), with parameters that mimic what a pedestrian might expect to see from motor vehicles while walking in an urban environment, we again can compare performance to normal vision, including phenomena like accumulation of information as successive motion cues are integrated, while also creating a baseline expectation for perceptual limits of specific devices.

## 7. Conclusion

Restoring motion perception through visual prostheses remains a significant challenge, with current devices demonstrating limited and variable success across individuals. While statistically significant improvements have been observed in many studies, absolute performance often falls short of levels required for functional tasks like navigation or object tracking. These discrepancies reflect both technological limitations and gaps in our understanding of how artificial vision engages the neural substrates of motion perception. In particular, the extent to which prosthetic stimulation activates the dorsal visual stream, beginning with area MT+, remains largely unexplored. As MT+ is central to motion integration and discrimination, clarifying how artificial stimulation recruits this area, and subsequent downstream areas, is critical to advancing the field.

A coherent picture of motion perception in prosthetic vision will require standardized methodologies, improved measurement of eye position, and task designs that probe higher-order processing. For engineers and designers, incorporating gaze contingency and scene-aware image filtering (e.g. depth, saliency, and thermal cues) may better approximate naturalistic vision. Researchers should prioritize experiments that quantify engagement of specific dorsal stream structures, including the suggested motion aftereffect and looming detection tasks. Clinicians must be prepared to communicate the distinction between statistical improvement and real-world functionality, while helping patients set expectations aligned with current device capabilities. Despite the current limitations, the trajectory of innovation across neural engineering, machine vision, and neuroscience suggests a future in which motion perception can be meaningfully restored above and beyond mere improvements in phosphene count. Achieving that goal depends on deepening our understanding of how artificial vision engages the brain’s motion-processing systems.

## Data Availability

No new data were created or analysed in this study.
